# Effect of awake prone position on diaphragmatic thickening fraction in patients assisted by noninvasive ventilation for hypoxemic acute respiratory failure related to novel coronavirus disease

**DOI:** 10.1186/s13054-021-03735-x

**Published:** 2021-08-24

**Authors:** Gianmaria Cammarota, Elisa Rossi, Leonardo Vitali, Rachele Simonte, Tiziano Sannipoli, Francesco Anniciello, Luigi Vetrugno, Elena Bignami, Cecilia Becattini, Simonetta Tesoro, Danila Azzolina, Angelo Giacomucci, Paolo Navalesi, Edoardo De Robertis

**Affiliations:** 1 Department of Medicine and Surgery, Università degli Studi di Perugia, Perugia, Italy; 2 Anestesia and Intensive Care Service 2, Azienda Ospedaliera di Perugia, Perugia, Italy; 3 Department of Medicine, Anesthesia and Intensive Care Clinic, Università di Udine, Udine, Italy; 4 Anesthesiology, Critical Care and Pain Medicine Division, Department of Medicine and Surgery, University of Parma, Parma, Italy; 5 Department of Medical Science, University of Ferrara, Ferrara, Italy; 6 Department of Medicine, University of Padova, Padova, Italy

## Abstract

**Background:**

Awake prone position is an emerging rescue therapy applied in patients undergoing noninvasive ventilation (NIV) for acute hypoxemic respiratory failure (ARF) related to novel coronavirus disease (COVID-19). Although applied to stabilize respiratory status, in awake patients, the application of prone position may reduce comfort with a consequent increase in the workload imposed on respiratory muscles. Thus, we primarily ascertained the effect of awake prone position on diaphragmatic thickening fraction, assessed through ultrasound, in COVID-19 patients undergoing NIV.

**Methods:**

We enrolled all COVID-19 adult critically ill patients, admitted to intensive care unit (ICU) for hypoxemic ARF and undergoing NIV, deserving of awake prone positioning as a rescue therapy. Exclusion criteria were pregnancy and any contraindication to awake prone position and NIV. On ICU admission, after NIV onset, in supine position, and at 1 h following awake prone position application, diaphragmatic thickening fraction was obtained on the right side. Across all the study phases, NIV was maintained with the same setting present at study entry. Vital signs were monitored throughout the entire study period. Comfort was assessed through numerical rating scale (0 the worst comfort and 10 the highest comfort level). Data were presented in median and 25th–75th percentile range.

**Results:**

From February to May 2021, 20 patients were enrolled and finally analyzed. Despite peripheral oxygen saturation improvement [96 (94–97)% supine vs 98 (96–99)% prone, *p* = 0.008], turning to prone position induced a worsening in comfort score from 7.0 (6.0–8.0) to 6.0 (5.0–7.0) (*p* = 0.012) and an increase in diaphragmatic thickening fraction from 33.3 (25.7–40.5)% to 41.5 (29.8–50.0)% (*p* = 0.025).

**Conclusions:**

In our COVID-19 patients assisted by NIV in ICU, the application of awake prone position improved the oxygenation at the expense of a greater diaphragmatic thickening fraction compared to supine position.

*Trial registration* ClinicalTrials.gov, number NCT04904731. Registered on 05/25/2021, retrospectively registered. https://clinicaltrials.gov/ct2/show/NCT04904731.

**Supplementary Information:**

The online version contains supplementary material available at 10.1186/s13054-021-03735-x.

## Introduction

Novel coronavirus-19 disease (COVID-19) outbreak has severely increased the hospitalizations for hypoxemic acute respiratory failure (ARF) requiring noninvasive ventilation (NIV) [1, 2]. This massive demand of ventilatory support has severely put in crisis the surge capacity response of intensive care unit (ICU) [3, 4], with potentially adverse consequences on clinical outcomes and mortality. In this scenario, awake prone position in combination or not with NIV has been considered to deal with COVID-19 patients admitted for hypoxemic ARF, in the hope of stabilizing respiratory status [5], avoiding intubation [6], and reducing overall proportion of severe COVID-19 progression to critical illness.

In non-COVID-19 acute respiratory distress syndrome (ARDS) patients undergoing invasive mechanical ventilation (IMV), prone position has been proven to improve gas exchange, lung aeration, and survival [7, 8]. However, despite the amelioration of oxygenation, also reported in awake patients with COVID-19-related ARDS [9, 10], maintaining prone position can be particularly challenging. In awake COVID-19 patients breathing with supplemental oxygen or assisted by NIV, poor comfort has been reported to be the main cause of short duration or discontinuation of prone position sessions [10–12].

It has been shown that prone position reduces chest wall flexibility [8]. If, one the one hand, this phenomenon allows a more homogeneous distribution of ventilation and regional lung stress in sedated and paralyzed ARDS patients undergoing IMV [13], on the other hand, the reduced chest wall compliance can cause discomfort and increase the work of breathing. According to the previous studies [14, 15], a reduced comfort is associated with both an increased work of breathing in invasively ventilated patients [14] and an increased diaphragmatic electrical activity in patients assisted through helmet NIV [15].

Diaphragmatic ultrasound has been used for assessing diaphragm activity both in hypercapnic [16] and hypoxemic ARF patients [17, 18] undergoing noninvasive respiratory support. In patients assisted by NIV for de novo hypoxemic non-COVID-19 ARF, a diaphragmatic thickening fraction < 36% predicted NIV failure [17]. We hypothesized that in ICU patients requiring NIV for COVID-19-related ARDS, the transition from supine to prone position may be responsible of an increase in diaphragmatic thickening fraction as a consequence of comfort modifications.

Our primary aim was ascertaining the effects of prone positioning on diaphragmatic thickening fraction. In addition, we assessed the effects of prone positioning on oxygenation, lung aeration, as assessed by lung sonography, breathing pattern, patient comfort, and hemodynamics. As additional endpoints, the effects exerted by body position on diaphragmatic thickening fraction were investigated after stratifying patients’ population for NIV outcome, i.e., failure versus success, within 48 h after study completion.

The present investigation is a secondary analysis of data prospectively collected to ascertain the characteristics and clinical course of COVID-19 patients in the ICU of the Perugia University Hospital, Italy, after approval by the local ethical committee (Protocol No. 3658/20).

## Methods

The present analysis was registered at www.clinicatrials.gov (NCT04904731). The study was conducted according to the principles outlined in the Helsinki Declaration, and written informed consent was waived owing to the observational nature of the study design, since all the patients were treated according to standard clinical practice.

### Patients

All adult patients, admitted to ICU with moderate-to-severe COVID-19-related ARDS [19] from February to May 2021, undergoing NIV combined to cycles of prone position (see Additional file 1 for awake prone position indications), as a rescue therapy, were considered eligible. Laboratory confirmation for infection by severe acute respiratory syndrome coronavirus 2 (SARS-CoV-2) was defined as a positive result of reverse transcriptase-polymerase chain reaction assay of naso-pharyngeal swabs obtained on hospital admission.

Exclusion criteria were: (1) any contraindications to NIV, (2) any contraindications to prone position [5], (3) dyspnea and/or tachypnea on study entry, (4) diagnosis of diaphragmatic palsy, (5) history of neuromuscular disease, (6) pregnancy, (7) impossibility to obtain a diaphragmatic and pulmonary ultrasound assessment (pneumothorax/pneumomediastinum with subcutaneous emphysema), (8) poor tolerance and/or severe worsening of clinical conditions during study phases necessitating NIV/prone position discontinuation, and (9) previous intubation during the same hospitalization period.

### Study protocol

On ICU admission, after NIV onset and before the first attempt of prone positioning, all eligible subjects underwent a standard clinical evaluation according to the current best clinical practice. According to our institutional protocol, NIV was set to provide a tidal volume (V_T_) on predicted body weight (PBW) ratio varying between 6 and 8 ml/kg with a combination of PEEP and inspired oxygen fraction (FiO_2_) to get a peripheral oxygen saturation (SpO_2_) between 92 and 96%. NIV was delivered through full face or oro-nasal facial mask connected via a double-tube circuit to a high-performance mechanical ventilator (Servo U Ventilator System, Getinge, Sweden). Thereafter, sonographic evaluations of the diaphragmatic thickening fraction and the lung aeration were performed in (1) supine position and (2) at 1 h following the application of prone position. Across the study trials, patients received no sedation or were sedated with dexmedetomidine (0.2–1.0 mcg/kg/h) alone or in combination with morphine (0.5–2 mg/h) to assure a Richmond Sedation Agitation Scale (RASS) score between 0 and − 1, according to institutional protocol, and the same level of sedation was maintained in supine and in prone position.

### Measurements and definitions

For each patients, the following data were recorded prior to study entry: age, gender, presence of comorbidities, arterial oxygen tension (PaO_2_) to FiO_2_ ratio, body mass index (BMI), PBW, NIV duration (days) preceding the trial. Vital signs, i.e., SpO_2_, EKG, and invasive arterial blood pressure, were continuously monitored throughout the entire study period. Expiratory V_T_ and respiratory rate were acquired by ventilator machine. Comfort and sedation were assessed through numerical rating scale (0 the worst comfort and 10 the highest comfort level) [15] and RASS [20] by physicians and/or nurses involved in the subject care.

At first, ultrasonographic evaluation of diaphragmatic thickening fraction and lung aeration were performed at bedside with subjects in a semi-recumbent position, using a portable ultrasound machine equipped with either a linear (7.5–12.0 MHz) or convex (2.0–4.0 MHz) probe (MylabX6, Esaote SPA, Italy).

With diaphragmatic ultrasound, diaphragmatic thickness was measured at both end-expiration and end-inspiration, and diaphragmatic thickening fraction, an indirect estimate of diaphragmatic effort [21–23], was calculated according to standard formula [24] as follows:

Thickening fraction (%) = (inspiratory thickness–expiratory thickness)/expiratory thickness * 100.

The diaphragmatic ultrasound was performed only on the right side due to the limitations offered by the spleen acoustic window and the acoustic barrier of air inside bowel and stomach on the left side [16]. After the first evaluation in supine position, a cutaneous marker was added to enhance the reliability of diaphragmatic sonography measurements by standardizing probe placement and minimizing measurement variability [16]. In each subject, three expiratory and inspiratory thickness measurements on the right side were acquired, and the mean values of expiratory and inspiratory thickness were computed and stored.

Lung aeration was evaluated through ultrasonography as previously described [25, 26], and a lung ultrasound score (LUS) was computed [25, 26]. A LUS ranging from 0 to 36 was obtained as the sum of the 6 regions scores on each hemithorax [25, 26].

Diaphragmatic and lung ultrasound were blindly performed and repeated at 1 h following the application of prone position by the same operator (GC), previously tested for intra- and interobserver variability of diaphragmatic ultrasound assessment [16, 27], who was not involved in patients care. Ultrasonographic and clinical data were gathered by a data collector, independent from the ultrasound operator and physicians involved in subjects’ care.

NIV failure, within 48 h following the study end, was defined as the recourse to IMV in response to the worsening in oxygenation associated or not with dyspnea and/or tachypnea onset (see Additional file 1 for NIV failure definition) [5, 28–31].

### Statistical analysis

Recent work reports a median diaphragmatic thickening fraction of 27% prior to CPAP onset in awake COVID-19 patients in supine position [18]. In the absence of data on diaphragm thickening fraction in awake COVID-19 patients undergoing NIV and prone positioning, we hypothesized that a mean 30% increase in diaphragmatic thickening fraction would occur after transition from supine to prone position, assuming a standard deviation of 10% in both conditions [18]. Accordingly, an overall sample size of 20 patients would be adequate for demonstrating an increase in diaphragmatic thickening fraction varying patient position from supine to prone, with type I error rate = 0.05, type II error rate = 0.20 and power 80%. Continuous variables were reported as median and interquartile range. Comparisons between supine and prone position were carried out by Friedman’s test for nonparametric repeated measures. A generalized linear mixed model was employed to ascertain the role of comfort in predicting diaphragmatic thickening fraction by adjusting for body position, i.e., supine versus prone. A mixed model with Satterthwaite methods of degrees of freedom was employed to assess the impact of body position on diaphragmatic thickening fraction in our patients, after stratifying study population according to NIV failure occurrence within 48 h following trial completion.

Two-tailed tests were applied for hypothesis testing, and statistical significance was considered for *p* values < 0.05. Statistical analyses were conducted using R3.5.2 software (The R foundation).

## Results

**Fig. 1:**
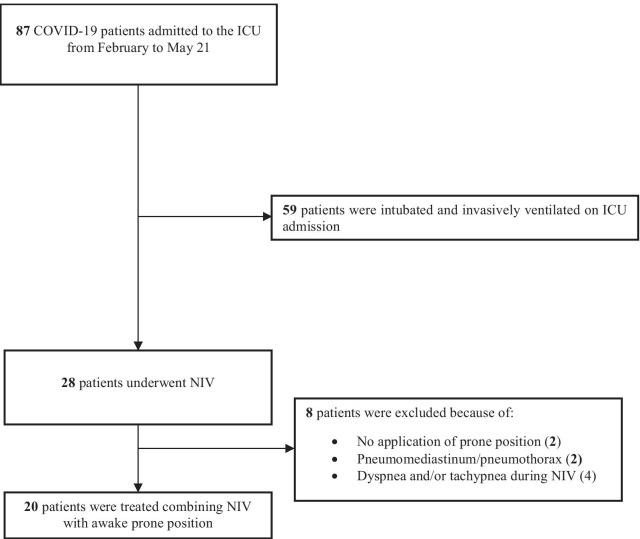
Enrollment flow diagram. COVID-19, novel coronavirus disease; ICU, intensive care unit; NIV, noninvasive ventilation

From February to May 2021, 87 patients were admitted to the ICU for hypoxemic acute respiratory failure COVID-19 related, of whom 59 required immediate IMV yet on ICU admission, whereas 28 patients were assisted by NIV (Fig. 1). In the subgroup of NIV patients, 20 subjects underwent cycles of awake prone positioning as a rescue ventilatory therapy (Fig. 1). In Table 1, the demographic characteristics of the study population at study entry are reported. In 14 patients, a PEEP ≥ 10 cmH_2_O was administrated with NIV.

**Table 1:** Patients’ characteristics at enrollment

Patients	Gender	Age	Comorbidities		BMI (kg/m^2^)	PBW (kg)	NIV before study enrollment (days)	Sedation	FiO_2_ (%)	PS (cmH_2_O)	PEEP (cmH_2_O)
1	F	68	Arterial hypertension		31.2	58.3	1		70	10	10
2	M	40	Other medical disease		26	70.6	3	Dexmedetomidine	60	10	10
3	M	54	–		26	66.0	2	Dexmedetomidine	40	10	8
4	F	60	–		27.7	61	2		50	10	10
5	M	71	History of cancer		24.7	75.1	4		55	6	10
6	F	57	Arterial hypertension		25.7	61.0	2		50	9	12
7	M	57	Arterial hypertension, dyslipidemia		38.5	80.0	1		50	5	12
8	M	46	–		24.7	83.5	1	Dexmedetomidine	45	5	8
9	M	79	Arterial hypertension, diabetes		31.2	64.9	2	Dexmedetomidine	45	5	9
10	M	75	Arterial hypertension		27.1	71.0	2	Dexmedetomidine	60	6	10
11	F	53	Other medical disease		29.5	61.5	1	Dexmedetomidine	100	8	8
12	M	73	Arterial hypertension, chronic kidney disease		31.9	65.0	2	Dexmedetomidine	50	6	10
13	F	71	Arterial hypertension, dyslipidemia, cardiovascular disease		44.9	51.9	2		60	12	10
14	M	62	Arterial hypertension, dyslipidemia, cardiovascular disease		27	63	1	Dexmedetomidine	65	5	8
15	M	51	Arterial hypertension, dyslipidemia		32.7	61.5	1		50	6	8
16	M	77	–		26.1	68.9	1	Dexmedetomidine	75	10	10
17	M	68	–		28	70	1	Dexmedetomidine + Morphine	50	10	10
18	M	68	–		31	72	1	Dexmedetomidine + Morphine	70	10	12
19	M	77	Arterial hypertension, cardiovascular disease, diabetes		29	69	1	Dexmedetomidine + Morphine	75	10	10
20	M	49	Arterial hypertension		28	70	1		40	8	10
Overall	16M/4F	65 (54–72)			28 (26–31)	68 (62–71)	2 (1–2)		53 (50–66)	9 (6–10)	10 (9–10)

BMI, body mass index; PBW, predicted body weight, NIV, noninvasive ventilation; FiO_2_, inspired oxygen fraction; PS, pressure support over PEEP; PEEP, positive end-expiratory pressure

The diaphragm and lung ultrasound variables are described in Table 2. Inspiratory diaphragmatic thickness and diaphragmatic thickening fraction increased moving from supine to prone position (inspiratory diaphragmatic thickness, *p* = 0.018; diaphragmatic thickening fraction *p* = 0.025). As expected, the application of awake prone positioning did not induce any modification in expiratory thickness of the diaphragm (*p* = 0.157). LUS slightly decreased with awake prone position application (*p* < 0.001).

**Table 2:** Diaphragm and lung ultrasound

Parameters	Supine	Prone	*p* value
*Diaphragmatic ultrasound*			
Inspiratory thickness (mm)	3.20 (2.68–3.63)	3.45 (2.92–4.03)	0.018
Expiratory thickness (mm)	2.40 (2.05–2.70)	2.45 (2.00–2.68)	0.157
Thickening fraction (%)	33.30 (25.70–40.50)	41.5 (29.80–50.00)	0.025
*Lung ultrasound*			
Lung ultrasound score	22.00 (20.00–24.30)	21.00 (16.80–23.00)	< 0.001

Data are presented as median and (25th–75th percentile)

*p* values refers to nonparametric Friedman test for repeated measures

Sedation and comfort as well as breathing pattern and hemodynamics are described in Table 3. RASS score did not change across the different study phases, whereas comfort score worsened with the awake prone position (*p* = 0.012). While V_T_ and V_T_/PBW decreased moving from supine to prone position (*p* < 0.001 for all comparisons), respiratory rate remained unchanged. As expected, SpO_2_ improved after prone position application (*p* = 0.008). Hemodynamics were not affected by prone position application.

**Table 3:** Sedation, comfort, breathing pattern, and hemodynamics

Variables	Supine	Prone	*p* value
*Sedation and comfort*			
RASS score	0.0 (− 0.3 to 0.0)	0.0 (0.0–0.0)	0.180
Comfort score	7.0 (6.00–8.00)	6.0 (5.00–7.00)	0.012
*Breathing pattern*			
V_T_ (ml)	493 (450–516)	453 (400–483)	< 0.001
V_T_/PBW (ml/kg)	7.41 (6.95–7.74)	6.65 (6.15–6.92)	< 0.001
Respiratory rate (breaths/min)	26 (24–28)	26 (22–30)	0.999
SpO_2_ (%)	96 (94–97)	98 (96–99)	0.008
*Hemodynamics*			
Systolic arterial blood pressure (mmHg)	131 (118–143)	128 (117–144)	0.491
Diastolic arterial blood pressure (mmHg)	71 (62–82)	74 (65–81)	0.819
Mean arterial blood pressure (mmHg)	89 (82–102)	93 (85–99)	0.346
Heart rate (beats/min)	62 (52–78)	64 (54–83)	0.251

RASS, Richmond Agitation-Sedation Scale; V_T_, tidal volume; PBW, predicted body weight; SpO_2_, peripheral oxygen saturation

Data are presented as median and (25th–75th percentile)

*p* values refers to nonparametric Friedman test for repeated measures

**Fig. 2:**
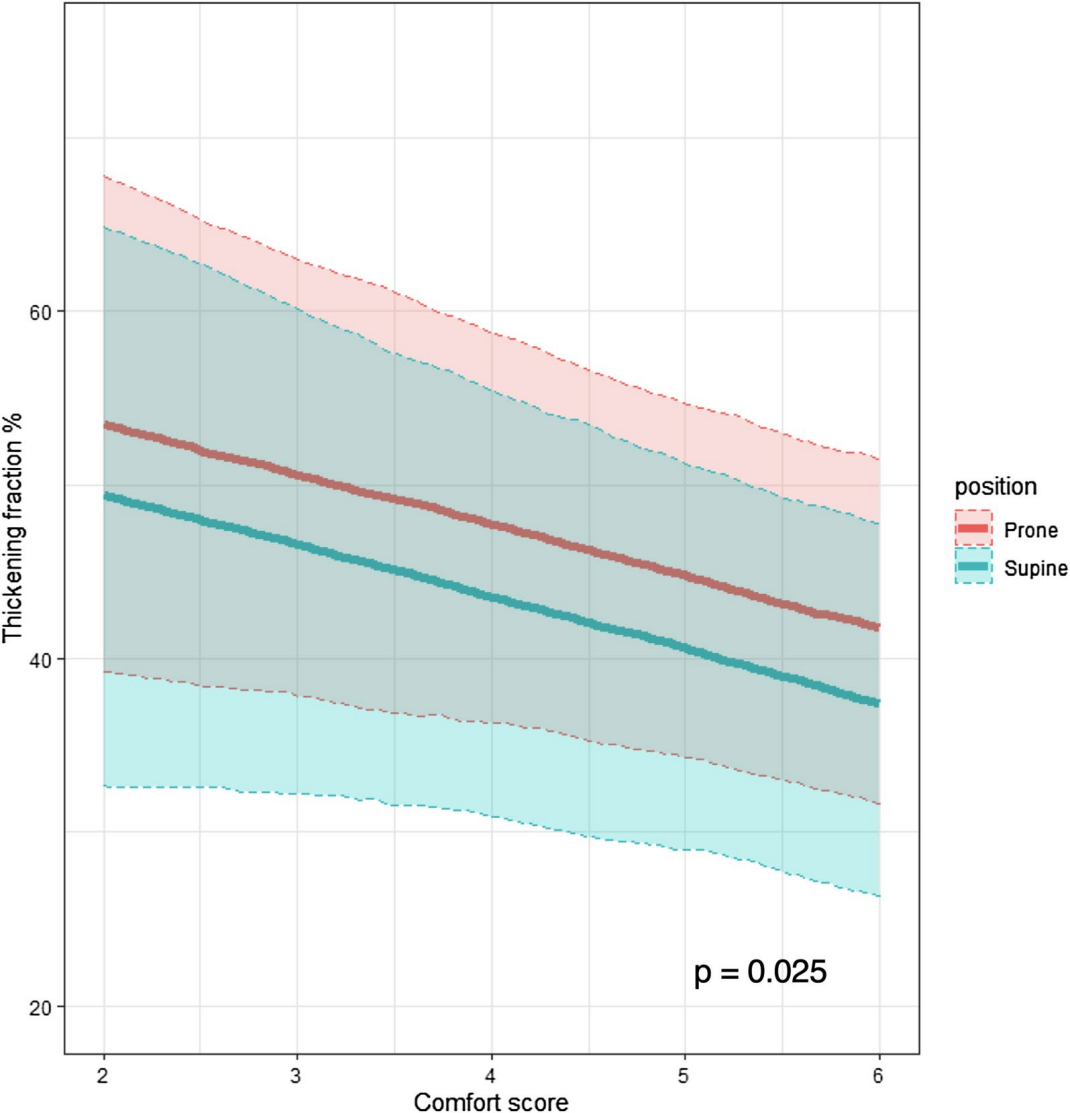
Generalized linear mixed model predicted diaphragmatic thickening fractions according to comfort scores, adjusted for body position, i.e., supine versus prone. Generalized linear mixed model predicted diaphragmatic thickening fractions according to comfort scores with 95% confidence intervals, adjusted for body position, i.e., supine (green) versus prone (red), are depicted. Fixed effect comfort score estimate (95% CI) =  − 2.9 (− 5.5 to − 0.4); *p* = 0.025

Figure 2 depicts the generalized linear mixed model predicted diaphragmatic thickening fractions according to comfort scores, adjusted for body position, i.e., supine versus prone. At decreasing comfort score corresponded an increasing in predicted diaphragmatic thickening fraction in both supine and prone position (*p* = 0.025).

**Fig. 3:**
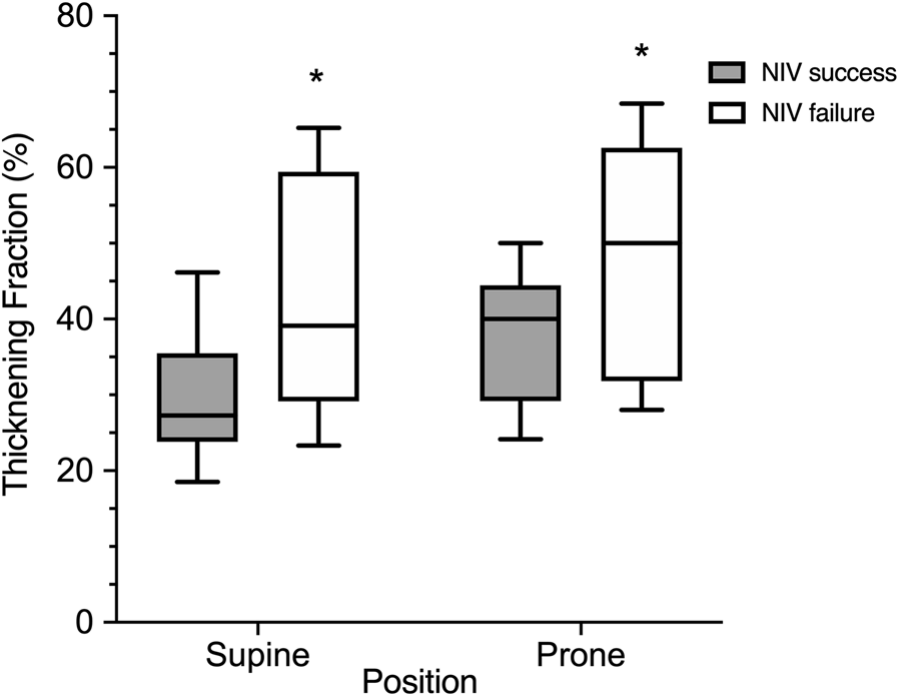
Impact of body position on diaphragmatic thickening fraction in the patients who experienced or not noninvasive ventilation failure within 48 h following study completion. Boxes and whiskers represent median, 25th–75th percentile, and minimum-to-maximum interval of diaphragmatic thickening fractions acquired in supine and prone position. Hollow boxes refer to patients who experienced NIV failure while grey boxes refer to patients with NIV success. *p = 0.008, NIV failure versus NIV success, refers to mixed model analysis carried out with Satterthwaite methods of degrees of freedom. NIV, noninvasive ventilation

Among the 20 patients enrolled in the study, nine experienced NIV failure and were intubated, within 48 h following study completion, because of the worsening of the oxygenation, in association or not with dyspnea and/or tachypnea onset. In Fig. 3, the impact of body position on diaphragmatic thickening fraction is plotted during NIV assistance, after stratifying study population according to NIV failure occurrence within 48 h after study end. Regardless of body position, the patients who experienced NIV failure showed a greater diaphragmatic thickening fraction compared to subjects who did not (*p* = 0.008).

## Discussion

The main findings of our study can be summarized as follows: (1) despite the improvement in oxygenation and the slight reduction in LUS, the application of awake prone position induced a more pronounced increase in diaphragmatic thickening fraction with respect to supine position; (2) the extent of the predicted diaphragmatic thickening fraction modification was inversely related to the degree of comfort achieved in supine and prone position; (3) irrespective of body position, the patients who experienced NIV failure were characterized by a greater diaphragmatic thickening fraction, as opposed to those who succeeded NIV.

Prone position is a rescue therapy applied in ARDS with the aim of improving gas exchange while improving lung mechanics [8]. Prone position promotes lung recruitment and gas redistribution as well as a more homogenous distribution of lung perfusion along the ventral-dorsal axis of the thorax [8]. When turning to prone position, however, all the physiological modifications of pulmonary ventilation and perfusion are achieved at the expense of chest wall compliance, which is primarily consequent to the limited expansion of the anterior and abdominal boundaries of the thorax on the bed surface [13, 32]. This phenomenon may reduce the comfort of awake patient laying in prone position with a consequent increase in inspiratory effort [14, 15].

Without improvement of lung mechanics in prone position, as reported in a previous investigation [13], the workload imposed on respiratory muscles throughout the respiratory cycle can be even more intense, with a definitively greater activation of the respiratory muscles, compared to supine position. Furthermore, once proned, patients are required to lie down in a well-defined position, so-called prone superman posture [12], also influenced by the presence of devices, such as NIV circuits and interfaces, vital signs monitoring apparatus, nasogastric tube for nutrition, infusion lines, and urinary catheter.

When turning patients from supine to prone position, we observed a reduction in comfort. Accordingly, we can speculate that the increase in diaphragmatic thickening fraction reported in our patients, was associated with the worsening of comfort due to reduction in chest wall flexibility in prone position. This is supported, on the one hand, by the decrease in V_T_ and V_T_/PBW observed in prone position compared to supine position, and, on the other hand, by the slight reduction in LUS that, despite statistically significant, was not so clinically relevant, suggesting a small modification in lung aeration at 1 h following prone position application. Moreover, in our setting, comfort confirmed an essential factor to deal with in course of NIV assistance overall. Indeed, in our series, a reduction in comfort was associated with an increase in predicted diaphragmatic thickening fraction both in supine and in prone position. This finding kept with previous reports [14, 15] indicating that a diminished comfort was associated with an increased work of breathing [14] and an increased electrical activity of the diaphragm [15].

In our patients, the boost of diaphragmatic thickening fraction induced by prone position was observed despite the SpO_2_ amelioration and the absence of respiratory rate modifications. This finding was in line with previous results [10], also obtained in COVID-19 patients, definitively showing an improvement of oxygenation with NIV without changes in respiratory rate in response to prone positioning [10].

Noteworthy, in COVID-19 patients, the control of breathing is particularly tricky due to the direct involvement of central neural nervous system by SARS-CoV-2 [33, 34] and the modifications of angiotensin-mediated sensitivity of the carotid bodies, expressing angiotensin-converting-enzyme 2 receptors [35]. Thus, in COVID-19 patients, the assessment of respiratory drive and inspiratory efforts are of pivotal importance in understanding the role played by patient-self-induced lung injury (PSILI) on disease progression [36].

The present study has several limitations. First, our study population was relatively small, but similar to the sample size of a recent investigation describing the impact of NIV on diaphragmatic thickening fraction in patients admitted with de novo hypoxemic ARF [17]. Second, the study population was not standardized for the COVID-19 ARDS phenotype or disease history and thus NIV might have been applied in patients with different lung involvements. Third, diaphragmatic and lung ultrasound were performed at baseline in supine position and after 1 h following prone position application, as also recently proposed in COVID-19 patients for sonographic assessment of lung aeration alone [37]. Different results might be obtained at different timepoints, i.e., 3–6 h from prone positioning onset as otherwise proposed [38]. Fourth, as ultrasound assessment is operator-dependent, the results obtained might be somewhat different among different operators. Fifth, as we did not collect arterial blood gases analysis during the study, we cannot provide data about arterial oxygen tension on inspired oxygen fraction ratio. Sixth, as we did not measure esophageal pressure, we are unable to determine the real impact of prone position on the chest wall mechanics. Finally, being the present study performed in a single ICU center during the third wave of COVID-19 pandemic, our results might be not generalized to different contexts and settings.

## Conclusions

In our cohort of COVID-19 patients, the combination of NIV and awake prone position overall improved oxygenation, while increasing diaphragmatic thickening fraction, compared to supine position. Our findings further contribute to point out the importance of inspiratory effort assessment in patients with COVID-19 hypoxemic ARF receiving NIV.

## Supplementary Information

**Additional file 1**. Indications for awake prone position onset during noninvasive ventilation; Noninvasive ventilation failure criteria.

## Data Availability

The data of the present investigation are available, on reasonable request, by contacting corresponding author.

## Abbreviations

ARDS: Acute respiratory distress syndrome
ARF: Acute respiratory failure
BMI: Body mass index
COVID-19: Novel coronavirus disease
FiO_2_: Inspiratory oxygen fraction
IMV: Invasive mechanical ventilation
LUS: Lung ultrasound score
NIV: Noninvasive ventilation
PaO_2_: Arterial oxygen tension
PBW: Predicted body weight
PSILI: Patient-self-induced lung injury
PEEP: Positive end-expiratory pressure
RASS: Richmond agitation sedation scale
SARS-CoV-2: Severe acute respiratory syndrome coronavirus 2
V_T_: Tidal volume
