# Supplementary material for: Effect of awake prone position on diaphragmatic thickening fraction in patients assisted by noninvasive ventilation for hypoxemic acute respiratory failure related to novel coronavirus disease
Source: Crit Care. 2021 Aug 24;25:305. doi: 10.1186/s13054-021-03735-x (PMC8383244; doi:10.1186/s13054-021-03735-x)
Supplement: Supplementary file 1 — Additional file 1. Indications for awake prone position onset during noninvasive ventilation; Noninvasive ventilation failure criteria. [file 13054_2021_3735_MOESM1_ESM.docx]

**Additional file 1**

Indications for awake prone position onset during non-invasive ventilation (NIV)

Prone position was applied in patients receiving NIV when they were hemodynamically stable, had peripheral oxygen saturation persistently <92% or PaO_2_/FiO_2_ <150 mmHg following the optimization of PEEP-FiO_2_ combination, in absence of dyspnea, tachypnea defined as a respiratory rate >35 breaths/min, signs of respiratory-muscle fatigue, and respiratory acidosis, as defined as pH <7.35.

Non-invasive ventilation (NIV) failure criteria

NIV failure was defined as the need for IMV due to ﻿worsening respiratory failure including at least 2 of the following: 1) unbearable dyspnea onset, 2) peripheral oxygen saturation < 90% during NIV for more than 5 minutes without technical disfunction, 3) signs of respiratory-muscle fatigue, 4) respiratory acidosis with pH < 7.35 despite NIV.

Also, patients were intubated in case of 1) cardiac or respiratory arrest; 2) inability to protect the airway; 3) coma or psychomotor agitation not controlled by continuous intravenous sedative infusion; 4) unmanageable secretions or uncontrolled vomiting; 5) life threatening arrhythmias or electrocardiographic signs of ischemia; 6) hemodynamic instability, defined as mean arterial pressure <60 mm Hg, despite fluids and or low dosage vasopressors administration; 7) intolerance to all interfaces for NIV.

The decision to proceed to intubation was always taken by the attending physician.
